# Stage-specific pharmacodynamic chloroquine and pyronaridine action on artemisinin ring-stage resistant Kelch C580Y mutation *Plasmodium falciparum* correlates to hemozoin inhibition process

**DOI:** 10.1128/aac.01208-24

**Published:** 2024-12-12

**Authors:** Abeer Sayeed, Revie Atkinson, Peter G. Vekilov, Jeffrey D. Rimer, David J. Sullivan

**Affiliations:** 1 Department of Molecular Microbiology and Immunology, Johns Hopkins Bloomberg School of Public Health 41531 , Baltimore, Maryland, USA; 2 Department of Chemical and Biomolecular Engineering, University of Houston 14743 , Houston, Texas, USA; 3 Department of Chemistry, University of Houston 165990 , Houston, Texas, USA; The Children's Hospital of Philadelphia, Philadelphia, Pennsylvania, USA

**Keywords:** hemozoin, chloroquine, pyronaridine, artemisinin, drug resistance

## Abstract

The antimalarial quinolines pyronaridine and chloroquine both inhibit hemozoin crystallization, predominately produced by *Plasmodium falciparum* intra-erythrocytic trophozoite stage parasites. Pyronaridine extends activity to ring-stage chloroquine-sensitive parasites, in contrast to chloroquine. Here, we investigated chloroquine and pyronaridine hemozoin inhibition type correlated to stage-specific activity on chloroquine-resistant ring-stage artemisinin sensitive and resistant *P. falciparum* CamWT and CamWT-C580Y parasites. Pyronaridine (2.8 μM) is tenfold more potent at beta-hematin nucleation than chloroquine (40 μM). Both pyronaridine and chloroquine (0.2 and 0.7 μM, respectively) had similar sub-μM inhibition of beta-hematin extension. *P. falciparum* CamWT-C580Y parasites produce smaller width hemozoin crystals which extend less than isogenic CamWT hemozoin. Stage-specific pulse dose pyronaridine and chloroquine on CamWT-C580Y or CamWT isogenic parasites observed 3- to 4-fold higher pyronaridine IC_50_s compared to 10- to 15-fold higher chloroquine on most CamWT-C580Y to CamWT stages. These findings collectively show that hemozoin nucleation inhibition widens stage-specific pyronaridine activity on *P. falciparum* drug-resistant parasites.

## INTRODUCTION

Currently, the artemisinins paired with mainly quinolines are rapidly parasiticidal even in the context of delayed parasite clearance to artemisinin combination therapy (ACT). Paradoxically, *Plasmodium falciparum* isolates from patients with delayed clearance have similar 72 h continuous IC_50_s values than those of the artemisinins (1). Pulsing artemisinins for 3–6 h on synchronized ring-stage parasites uniquely differentiated elevated pulse artemisinin ring-stage IC_50_s in the delayed clearance *P. falciparum* isolates (2) which were later correlated to mutations predominately in the propeller region of the *P. falciparum* Kelch 13 gene (3, 4).

The ring-stage resistant *P. falciparum* Kelch 13 mutant isolates manifest a delayed ring phenotype lasting 6–12 h longer than artemisinin ring-stage sensitive isolates (5). Roepe and colleagues carefully quantified free heme and hemozoin in artemisinin-sensitive CamWT parasites and the isogenic-resistant isolates CamWT-C580Y parasites noting minimal free heme in CamWT with a burst of free heme to 4 mM around trophozoites at 32 h post-invasion (6). In contrast, CamWT-C580Y had nearly 1 mM free heme slowly rising to 30 h peak at 1.3 mM (6). The total hemozoin was similar with CamWT-C580Y being slightly higher at 350 mM compared to CamWT at 250 mM. In another study, Sun and colleagues, noted in 3D7-C580Y parasites a reduction in hemozoin product at 10 nmol compared to 15 nmol in 3D7 parasites with slower growth and lower number of progeny merozoites in the 3D7-C580Y parasites (7).

Tilley and colleagues noted heightened sensitivity with lower pulse artemisinin concentrations at early 0–3 h ring stage, followed by higher pulse artemisinin concentrations during ring stages with lower pulse drug IC_50_s at trophozoite stages (8), which was later mechanistically correlated to burst of hemoglobin digestion at early ring, followed by minimal hemoglobin catabolism until trophozoite stages with return of more sensitive short artemisinin pulses (9). The new piperaquine survival assay used a 48 h drug “pulse,” followed by 24 h off to show higher piperaquine survival rates in those recrudescent clinical isolates after ACT with dihydroartemisinin-piperaquine (10). Another lumefantrine/artemether “pulse” assay dosed at 12 h intervals for the 48 h cycle indicating synergy with artemether from hour 0–12 h in artemether sensitive and resistant *P. falciparum* isolates as well as lower lumefantrine alone IC_50_s at 0–12 h with a near doubling the next 12 h in both isolates (11).

As members in the 4-amino quinoline drug class, pyronaridine and chloroquine bind heme, localize to the digestive vacuole as weak bases and inhibit heme crystal formation (12, 13). The structure of chloroquine superimposes upon pyronaridine (14). Here, we measured these quinolines for inhibition in a lipid-based heme crystal nucleation assay (15) as well as extension from both performed beta-hematin (16), CamWT and CamWT-C580Y parasite-derived hemozoin. We also looked at short 3–6 h stage-specific pulse drug pyronaridine and chloroquine on *P. falciparum* CamWT and CamWT-C580Y parasites.

These experiments collectively show that hemozoin nucleation inhibition, in addition to hemozoin extension or growth inhibition, widens stage-specific pyronaridine activity on *P. falciparum* drug-resistant parasites. This may pertain to early onset of sufficient hemoglobin catabolism in C580Y ring-stage parasites compared to CamWT.

## RESULTS

### Beta-hematin nucleation and extension

**Fig 1:**
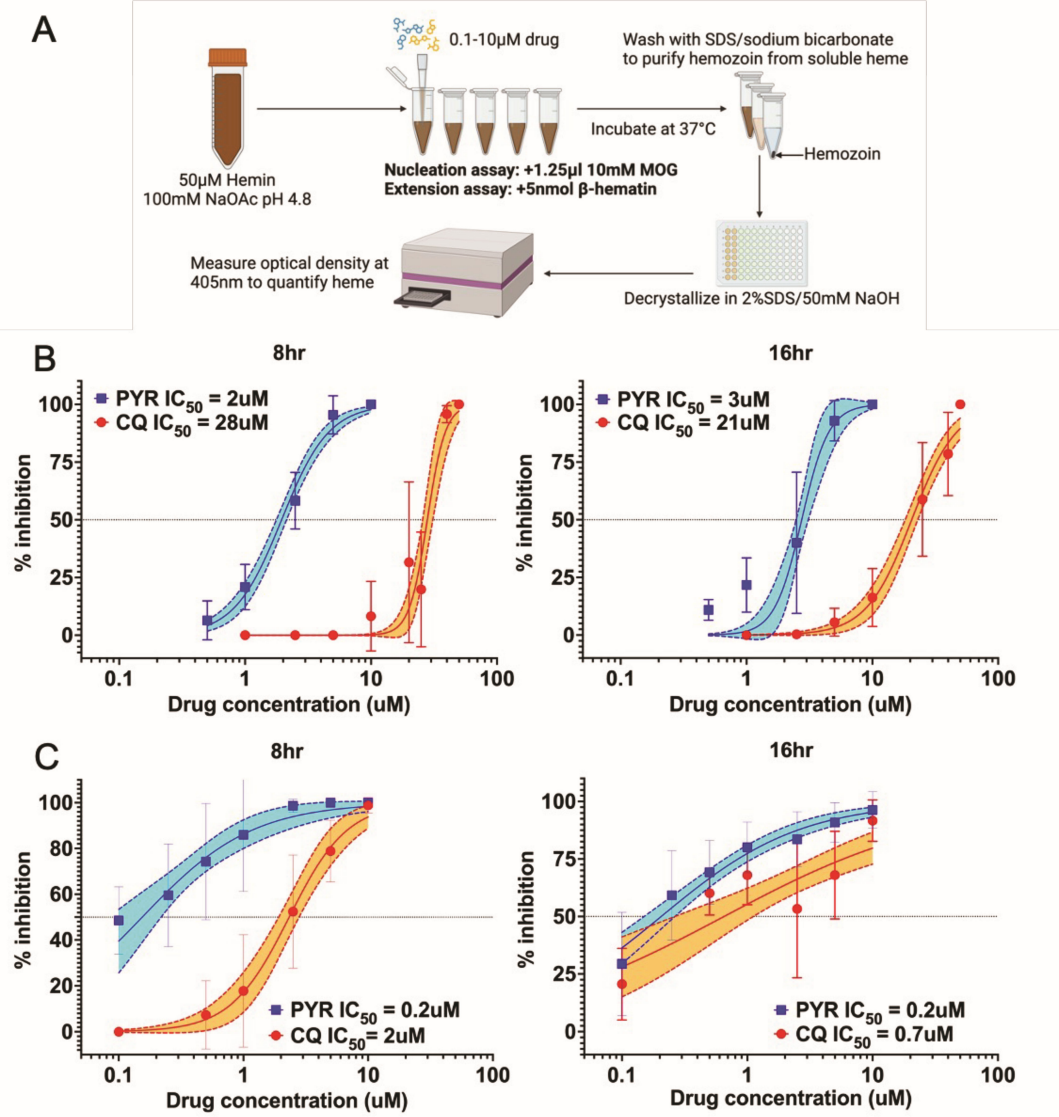
Beta-hematin nucleation and heme crystal extension inhibition. (**A**) Nucleation assays were 50 μM MOG and hemin incubated in 250 μL acetate pH 4.8 for 8 and 16 h in technical triplicate with biologic replicates *n* = 4 for 8 h and *n* = 5 for 16 h incubations. (**B**) Extension assays were 50 μM hemin seeded with 5 nmol beta-hematin in 250 μL for 8 and 16 h in technical triplicate with biologic replicates *n* = 4 for 8 h and *n* = 7 for 16 h incubations. (**C**) Pyronaridine inhibited hemozoin nucleation at 8 and 16 h with 2 and 2.8 μM IC_50_, while chloroquine achieved similar outcomes with 28 and 21 μM, respectively. Both pyronaridine and chloroquine at 8 and 16 h for heme extension have IC_50_ within 1 μM. IC_50_ values calculated from non-linear regression curves of % inhibition plotted values are reported on graph along with shaded regions depicting 95% confidence intervals of regression models.

Submicroscopic hemozoin is nucleated at late ring stages with predominate extension and some nucleation at trophozoite stages. In the lipid (monooleylglycerol-MOG) initiated beta-hematin nucleation assay with 12.5 nmol heme input, the no drug inhibition product was consistently 5.2 nmol at 8 h and 5.9 nmol at 16 h. Pyronaridine is 10-fold more potent in beta-hematin nucleation inhibition at both 8 and 16 h than chloroquine (Fig. 1A). The beta-hematin extension assay incubates 5 nmol of preformed beta-hematin with 12.5 nmol of hemin in 250 mL with typically 2–2.4 nmol heme extension. While pyronaridine (0.2 μM) was more than 10-fold more potent than chloroquine (2.3 μM) at 8 h the fold change was similar at 3-fold measured at 16 h between pyronaridine (0.2 μM) and chloroquine (0.7 μM) (Fig. 1B).

### *P. falciparum* hemozoin in CamWT-C580Y

**Fig 2:**
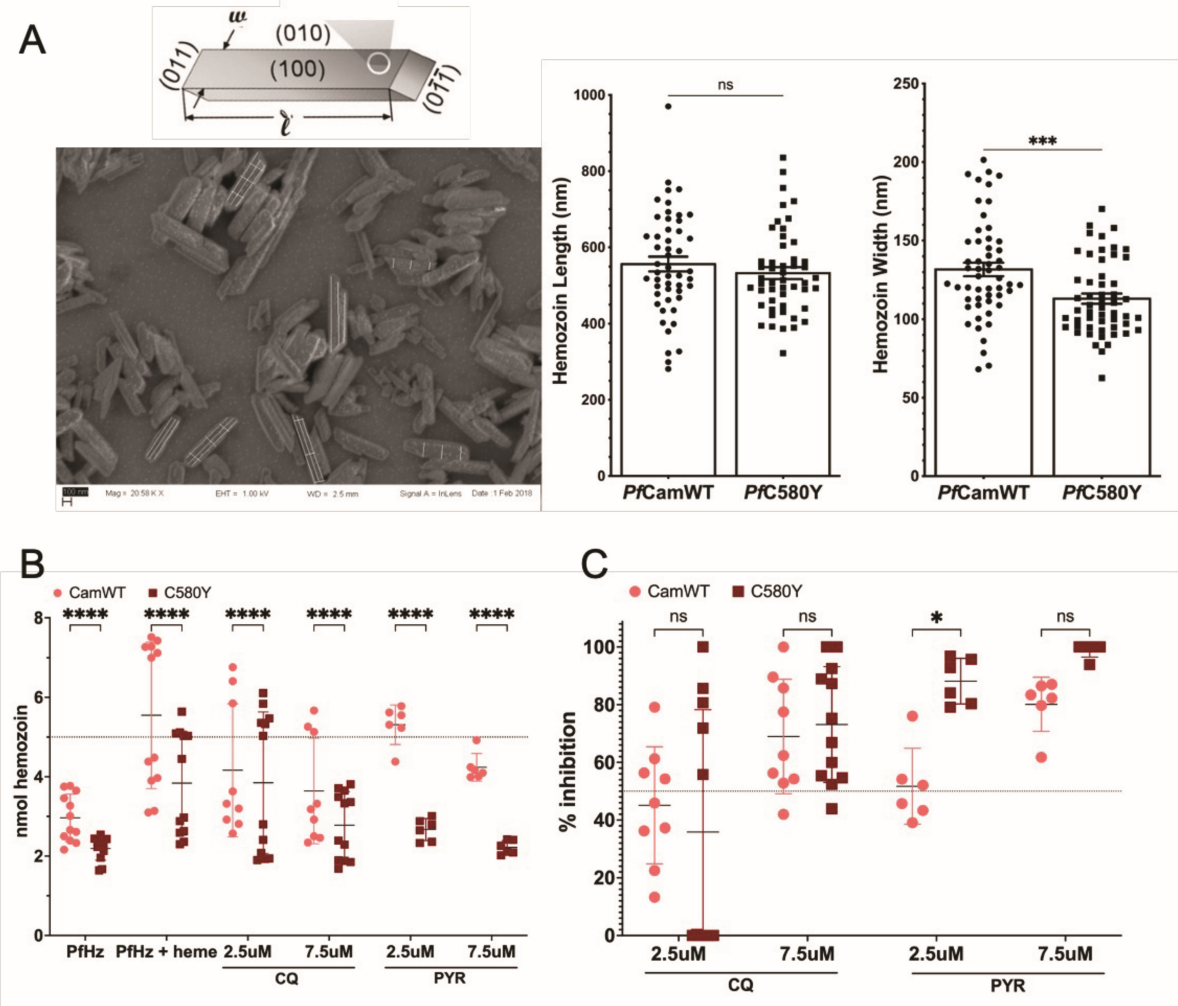
Purified hemozoin from CamWT and C580Y parasites. (**A**) C580Y parasites produce smaller-sized hemozoin crystals in width compared to CamWT in unpaired *t* test. (**B**) Hemozoin from *Pf*C580Y extends significantly less than *Pf*CamWT and (C) pyronaridine (PYR) has greater inhibition for heme crystal formation than chloroquine.

While total hemozoin in artemisinin ring-stage resistant *P. falciparum* isolates is similar, we investigated size differences. CamWT-C580Y has similar length of around 550 nm to CamWT, but has smaller width at 120 nm versus CamWT at 140 nm (Fig. 2A). We measured the difference in heme crystal extension from preformed hemozoin from both isolates with 2.5 nmol extended with CamWT hemozoin, but only 1.8 nmol with CamWT-C580Y (Fig. 2B) with greater inhibition of heme crystal extension with pyronaridine versus chloroquine (Fig. 2C)

### Pulse drug stage-specific inhibition

**Fig 3:**
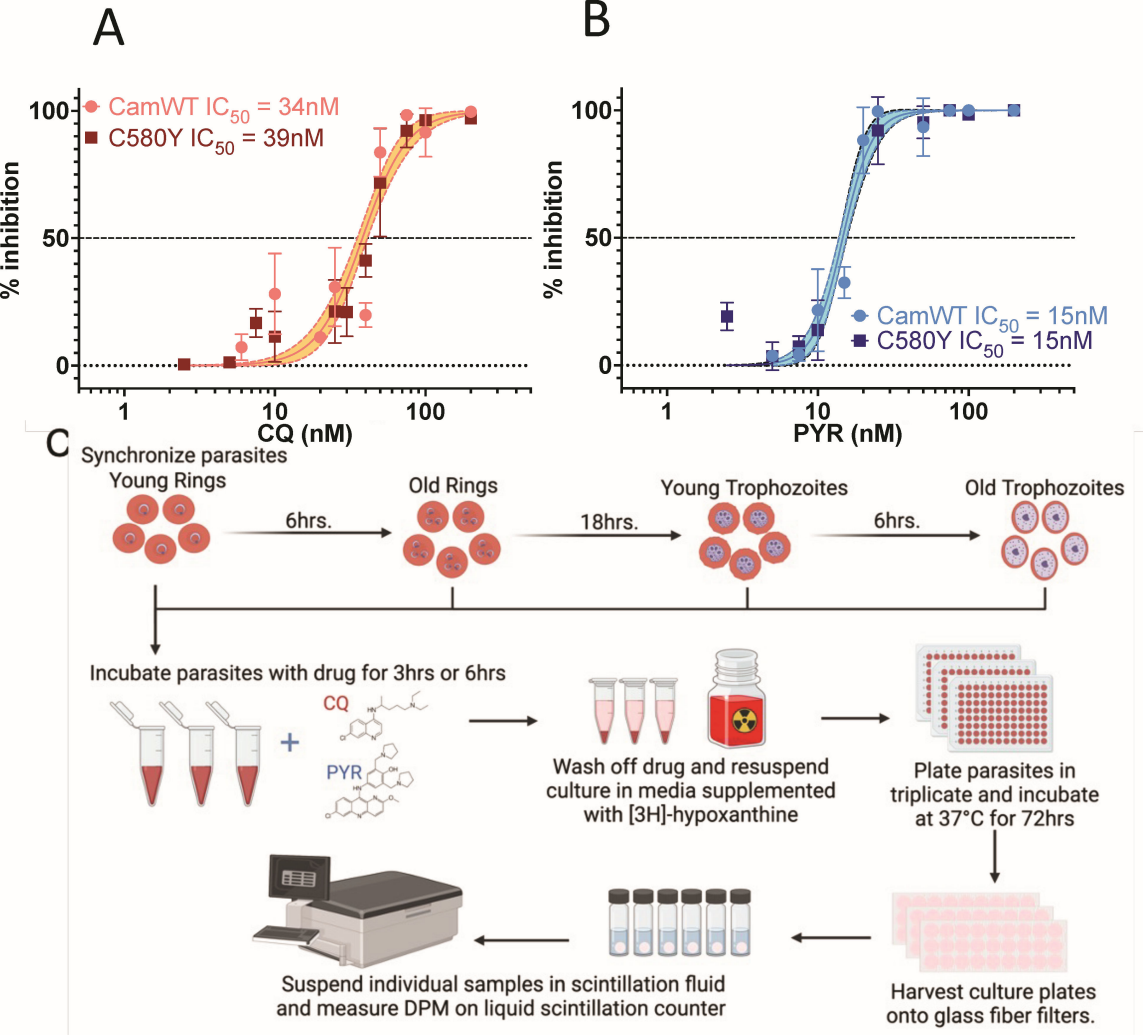
Continuous 72 h (A) chloroquine (CQ) and (B) pyronaridine (PYR) IC_50_. Assays performed in biologic triplicates with technical triplicates. (C) Design of stage-specific 3 and 6 h pulse drug experiments.

**Fig 4:**
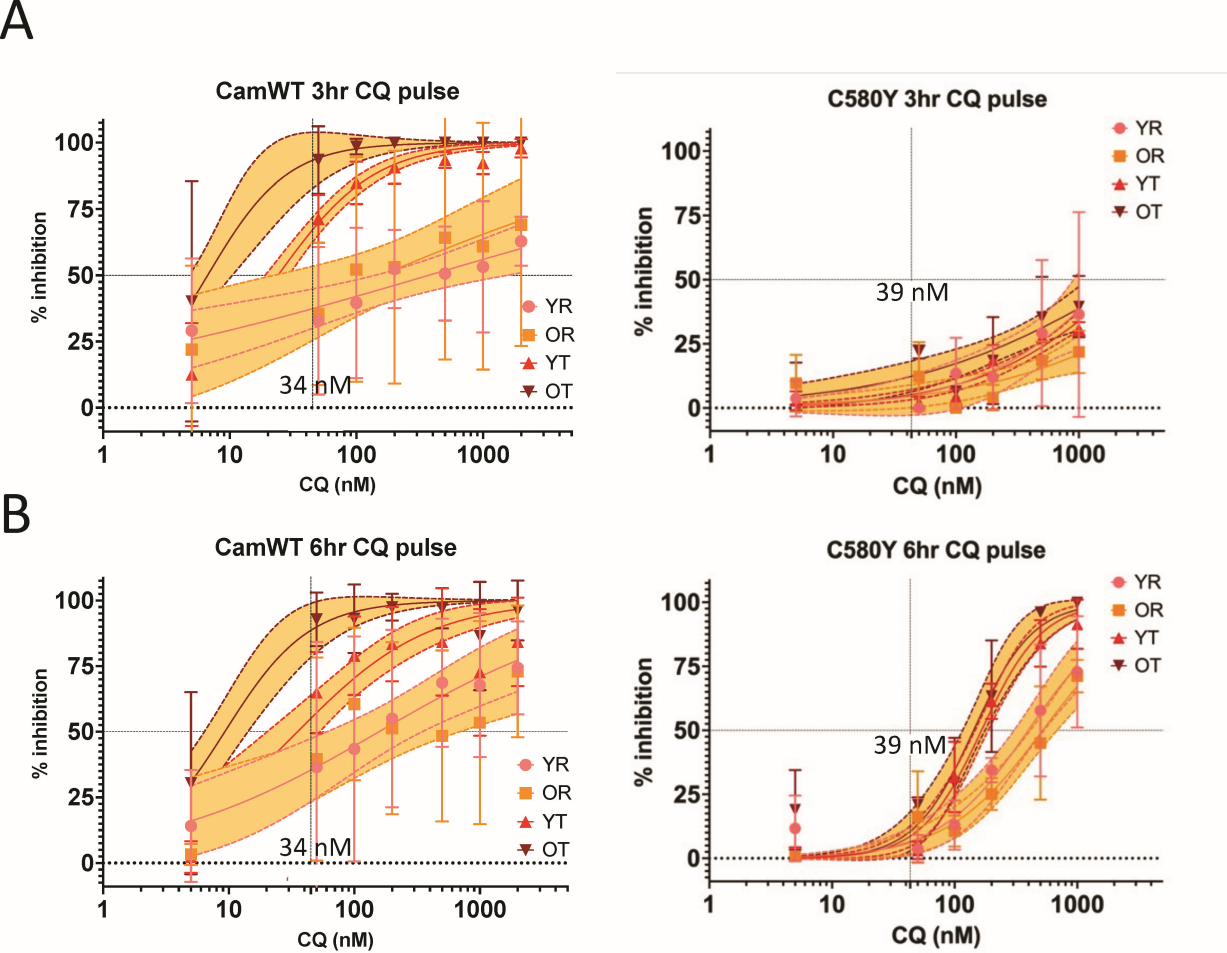
Pulse chloroquine IC_50_s. *P. falciparum* isogenic isolates CamWT and C580Y were treated with 3 or 6 h drug pulses at young rings (YR), old rings (OR), young trophozoites (YT), and old trophozoites (OT). The CamWT and C580Y and 3 and 6 h chloroquine (CQ) pulses were done in biologic triplicate with technical triplicates. IC_50_ values calculated from non-linear regression curves of % inhibition plotted values are reported along with shaded regions depicting 95% confidence intervals of regression models.

**Fig 5:**
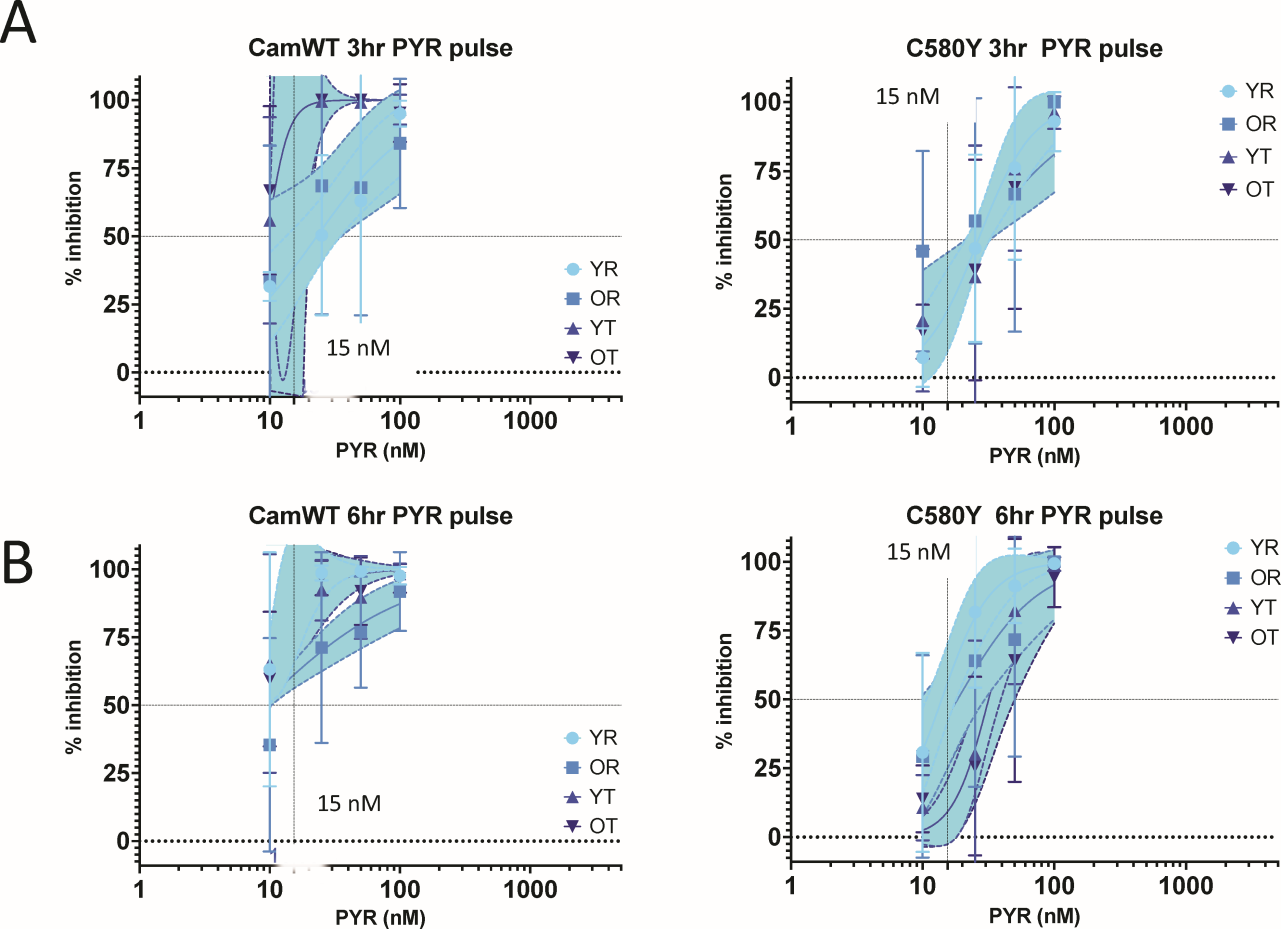
Pulse pyronaridine IC_50_s. *P. falciparum* isogenic isolates CamWT and C580Y were treated with 3 or 6 h drug pulses at young rings (YR), old rings (OR), young trophozoites (YT), and old trophozoites (OT). The CAMWT and C580Y 3 and 6 h pyronaridine (PYR) pulses were done in biologic triplicates with technical triplicates. IC_50_ values calculated from non-linear regression curves of % inhibition plotted values are reported along with shaded regions depicting 95% confidence intervals of regression models.

The continuous 72 h IC_50_s were observed to be similar for chloroquine at approximately 34–39 nM for both CamWT and C580Y isolates. Likewise, the continuous IC_50_s for pyronaridine approximated 15 nM (Fig. 3A and B). Pulse drug exposure for 6 or 3 h noted divergent stage-specific inhibition (Fig. 3C). On CamWT ring-stage parasites, seven times the 6 h pulse dose chloroquine concentration for trophozoite IC_50_ (180 nM compared to 35 nM) was required (Fig. 4; Table 1), while 10 nM pyronaridine achieved the IC_50_ at all four stages-young rings, old rings, early trophozoites or late trophozoites/schizonts (Fig. 5; Table 1). In contrast, we observed for C580Y parasites, that the 6 h pulse chloroquine concentration to achieve ring-stage IC_50_ is approximately 10 times the continuous IC_50_ and was triple the trophozoite IC_50_s. Pyronaridine demonstrated greater ring-stage inhibition with lower IC_50_ at both early and late ring-stage pulse pyronaridine.

**TABLE 1:** Pulse dose IC_50_ (nM) with 95% confidence intervals^*a*^

	CQ CamWT IC_50_ (95% CI)	CQ C580Y IC_50_ (95% CI)	PYR CamWT IC_50_ (95% CI)	PYR C580Y IC_50_ (95% CI)
YR 3 h	371 (141–1,562)	1,864 (899–38,467)	19 (15–24)	28 (22–35)
OR 3 h	156 (26–722)	11,378 (2,081 to NA)	17 (0–36)	15 (2–29)
YT 3 h	24 (19–29)	2,559 (1,862–4,014)	10 (7–11)	29 (19–40)
OT 3 h	7 (5–10)	2,580 (1,123–23,614)	10 (NA to 11)	30 (21–41)
YR 6 h	157 (66–342)	398 (305–532)	9 (4 to NA)	14 (11–18)
OR 6 h	232 (89–683)	522 (415–679)	15 (7–23)	18 (9–29)
YT 6 h	35 (23–52)	166 (147–188)	6 (1–10)	32 (26–39)
OT 6 h	9 (5–14)	143 (116–175)	9 (6–10)	38 (28–49)

^*a*^ CamWT and C580Y were treated with 3 or 6 h drug pulses at young rings (YR), old rings (OR), young trophozoites (YT), and old trophozoites (OT).

The 3 h drug pulses magnified these differences for chloroquine. For instance, the young ring chloroquine pulse dose Cam WT IC_50_ at 371 (95% CI = 141–1,562) nM is 11 times the continuous IC_50_. The C580Y isolate showed high 2,000 (YR, YT, and OT) to 10,000 (OR) nM pulse chloroquine IC_50_s with wide 95% CI. Pyronaridine with 3 h pulse dose concentrations remained in the 10–15 nM IC_50_ for CamWT. Against the C580Y, the pyronaridine pulse IC_50_s on both ring stages was near 15–28 nM while trophozoite was ~30 nM.

## DISCUSSION

Antimalarial quinolines differ in type of heme crystal inhibition on three distinct growing faces (17). While the 4-amino quinoline structure of chloroquine is superimposable on pyronaridine, pharmacodynamics and drug resistance differ. Here, we observed distinct patterns of heme crystal nucleation inhibition manifest by almost 10-fold more pyronaridine potency compared to chloroquine. The heme crystal extension inhibition was similar.

In the ring stage artemisinin-resistant C580Y isolate, characterized by a phenotypic increased cytosolic stress response, hemoglobin uptake is altered in timing and rate while the total catabolized is similar in isogenic *P. falciparum* isolates for C580Y. Here, we noted smaller width to C580Y heme crystal which suggests more nucleation centers in C580Y isolates compared to CamWT. Measurement of heme crystal extension observed slightly smaller amounts in the C580Y compared to CamWT. C580Y may utilize more numerous nucleation centers to equalize heme detoxification via heme crystallization.

Probing synchronized *P. falciparum in vitro* culture stage-specific action with short drug pulses over the 48 h cycle extends pyronaridine to early and late ring stages with little difference on 3 and 6 h pulses. Chloroquine required higher drug concentrations in C580Y isolates for 3 h drug pulses at all stages and ring stages of CamWT. Pyronaridine was more potent at ring stages of C580Y with lower IC_50_ compared to trophozoites which, according to Heller and Roepe (6) experiments mapping C580Y hemoglobin catabolism, has ring-stage hemoglobin degradation with possible nucleation heme crystallization centers higher than CAMWT at rings. In CamWT parasites, pyronaridine was essentially equally potent in nM drug concentration achieving IC_50_ for all stages. In a previously published single-dose malaria mouse model, both single-dose pyronaridine (10–250 mg/kg) and three daily doses of artesunate (200 mg/kg) had similar million-fold (log 6) 48 h parasite reduction, while 250 mg/kg chloroquine and amodiaquine (amodiaquine’s molecular structure is also superimposable on pyronaridine) at 250 mg/kg had a 1,000-fold (log 3) parasite reduction (18).

These experiments show that hemozoin nucleation inhibition widens stage-specific pyronaridine activity on *P. falciparum* drug-resistant parasites. This potentially might augment the ring-stage killing action when used in combination with the artemisinins, especially in the setting of ring stage artemisinin partial resistance appearance in Africa (19).

## MATERIALS AND METHODS

### Stock preparation

Chloroquine diphosphate salt (CQ) (Sigma C-6628) was dissolved in sterile cell-culture grade H_2_O to make a 10 mM stock. Pyronaridine tetraphosphate (PYR) (Sigma P0049) was dissolved in sterile cell-culture grade H_2_O to make a 10 mM stock. All drugs were further diluted either in H_2_O for hemozoin nucleation and extension assays or cell culture medium for parasite assays. Hemin chloride (CHEM-IMPEX 00813) was dissolved in DMSO to make a 10 mM stock. It was further diluted in 100 mM sodium acetate (pH 4.8) to make a 50 μM working solution for hemozoin nucleation and extension assays. 1-Oleoyl-rac-glycerol (MOG) (Sigma M7765) was dissolved in 100% ethanol to make a 10 mM stock. β-hematin was previously synthesized and diluted in H_2_O.

### Hemozoin nucleation assay

Hemin chloride in 100 mM sodium acetate (pH 4.8) was prepared fresh at 50 μM from a 10 mM hemin stock in DMSO. For each reaction, up to 250 μL of 50 μM hemin and 1.25 μL of 10 mM MOG were added to a 1.5 mL Eppendorf tube. For samples with drug, up to 10 μL of CQ or PYR was added to each reaction for a range of final drug concentrations at 0.5–50 μM. Positive controls included 50 μM hemin and 50 μM MOG only to measure nucleation in the absence of drug inhibition. Negative controls included 50 μM hemin only to account for background levels of heme after washing. Each sample was run in triplicate tubes. Tubes were vortexed and incubated in a 37°C water bath for 8, 16, or 48 h. After incubation, 10 μL of 10% SDS was added to each tube. Formed hemozoin crystals were isolated with a series of washes: first with 250 μL of 2% SDS/80 mM sodium bicarbonate pH 9.1 and sonication, followed by 250 μL of 2% SDS and sonication. The remaining pellets were de-crystallized using 250 μL of 2% SDS/50 mM NaOH at room temperature for 30 min, followed by heme quantification by spectroscopy. Full method is graphically depicted in Fig. 1A.

### Hemozoin extension assay

Hemin in 100 mM sodium acetate (pH 4.8) was prepared fresh at 50 μM from a 10 mM hemin stock in DMSO. For each reaction, up to 245 μL of 50 μM hemin and 5 μL of 1 nmol/ μL β-hematin was added to a 1.5 mL Eppendorf tube. For samples with drug, up to 10 mL of CQ or PYR was added to each reaction for a range of final drug concentrations at 0.1–10 μM. Positive controls included 50 μM hemin and 5 nmol β-hematin only to measure extension in the absence of drug inhibition. Negative controls included 5 nmol of β-hematin in sodium acetate only (without hemin) to account for any loss in β-hematin after washing. Each sample was run in triplicate tubes. Tubes were vortexed and incubated in a 37°C water bath for 8, 16, or 48 h. After incubation, 10 μL of 10% SDS was added to each tube. Extended hemozoin crystals were isolated with a series of washes: first with 250 μL of 2% SDS/80 mM sodium bicarbonate and sonication, followed by 250 mL of 2% SDS and sonication. The remaining pellets were de-crystallized using 250 μL of 2% SDS/50 mM NaOH at room temperature for 30 min, followed by heme quantification by spectroscopy. The full method is graphically depicted in Fig. 1B.

### Hemozoin quantification

The de-crystallized samples were briefly sonicated before quantification. In total, 200 μL of each sample was plated in a clear, flat-bottom 96-well plate. A standard curve was developed by creating a serial dilution of hemin in 2% SDS/50 mM NaOH at concentrations ranging from 1 to 10 nmol hemin per well. About 200 μL 2% SDS/50 mM NaOH was plated as a negative control background measure for quantification. Both the standard curve and negative controls were plated in duplicate. The plate was read on the BioTek Synergy H1 plate reader to measure absorbance at a wavelength of 405 nm. The lower limit of detection was determined to be at an optical density (OD) of 0.2 and saturation occurred at an OD of 2.5. Hemozoin samples were quantified by using a linear regression equation of the hemin standard curve to calculate the total amount of heme per well.

### *P. falciparum* parasite culture maintenance

*P. falciparum* parasite cultures were maintained *in vitro* using a method modified by Trager and Jensen (20). Parasites were cultured in RPMI 1640 medium supplemented with l-glutamine, 25 mM HEPES, 0.25% NaHCO_3_, 0.37 mM hypoxanthine, 50 μL of 50 mg/mL gentamycin, and 10% human serum. Cultures were incubated at 37°C in 5% CO_2_/5% O_2_/balanced N_2_. Fresh RBCs were used to maintain cultures at 2–4% hematocrit. Blood was obtained from living donors under Johns Hopkins IRB-approved protocols. *P. falciparum* CamWT (MRA-1250) and *P. falciparum* CamWT_C580Y (MRA-1251) were generously contributed by David A. Fidock.

### Hemozoin isolation from culture

*P. falciparum* cultures were expanded to 100–200 mL volumes at 4% hematocrit and high (5–10%) parasitemia. Parasites were isolated after centrifuging the cultures and treating the RBC pellet volume-to-volume with 2 mg/mL saponin in a 37°C water bath for 10 min. Once RBC lysis had occurred, parasites were washed with 50 mL of 1× PBS and centrifuged at 3,000 rpm for 10 min. The parasite pellet was resuspended in 1 mL of 5 mM sodium phosphate (pH 7.5) and transferred to a 1.5 mL Eppendorf tube. It was then sonicated and centrifuged at 13,000 rpm for 5 min. The supernatant was removed, and the pellet was flash-frozen on dry ice for 10 min. Once thawed, the pellet was washed once more in 5 mM sodium phosphate with sonication. A final wash with water was performed and the pellet was resuspended in 200–500 μL H_2_O for quantification. During quantification, a spectral scan was performed measuring at a range of absorbances between 390 and 430 nm to ensure heme presence at 405 nm. Hemozoin stocks were normalized to 1 nmol/mL in H_2_O.

### Field emission In-lens scanning electron microscope imaging

Hemozoin was coated on silica chips following a previously described protocol (11). About 7–8 nmol hemozoin was coated on each chip. Hemozoin coating was verified using microscopy. After critical point drying, the hemozoin containing chips were coated with gold and imaged on the FEISEM (Leo 1550). About 15–20 images were taken per sample.

### Hemozoin measurements

The image analysis software ImageJ was used to measure hemozoin dimensions. Images were magnified so that roughly 20 pixels was equivalent to 100 nm. The line-drawing tool was used to measure the scale on each image to produce a pixel equivalent value. The line-drawing tool was then used to measure 3 lengths and 3 widths per crystal as depicted in Fig. 2A. The software automatically converted the measurements to nm, which were then averaged to produce one measurement of length and one measurement of width per crystal.

### 72 h-continuous drug inhibition assay

*P. falciparum* cultures were synchronized to ring stage by adding 5 volumes of 5% sorbitol to the RBC pellet after centrifugation. Cultures were incubated in sorbitol at room temperature for 10 min, after which they were washed with 1× PBS. Parasitemia was assessed by microscopy of a Giemsa-stained blood film. 72 h drug inhibition was assessed using a modified [3H]-hypoxanthine uptake assay (21). Cultures were plated in a tissue-culture treated 96-well plated at 0.6% parasitemia, 2% hematocrit, in [3H]-hypoxanthine supplemented complete media treated with a range of drug concentrations in triplicate. Negative control growth wells contained 100 µM CQ. Positive control growth wells contained drug-free medium. Drugs were plated from 10× stocks and were diluted 1:10 in each well with a final culture volume of 200 μL/well. The final concentration of [3H]-hypoxanthine was 0.5 μCi/well. Plates were incubated under special gas conditions (5% CO_2_/5% O_2_/balanced N_2_) at 37°C for 72 h after which they were frozen at −80°C for at least 24 h. Plates were thawed at 37°C for 1 h and harvested onto glass fiber filters (Brandel GF/C) using a cell harvester (Brandel MB48). Individual samples were resuspended in liquid scintillation fluid and [3H]-hypoxanthine incorporation was measured in disintegrations per minute (DPM) on a liquid scintillation counter.

### Pulse-drug inhibition assay

Parasite cultures were double-synchronized to ring stage and parasitemia was assessed as described previously. Stage-specific assays were performed at young rings (post-synchronization), old rings (6 h post-synchronization), young trophozoites (24 h post-synchronization), and old trophozoites (30 h post-synchronization). At each stage, cultures were treated with drug at a range of concentrations (5 nM to 2 µM) for either 3 or 6 h, after which drug was washed off three times with incomplete media. Positive controls with no drug treatment were included. Washed parasites were plated in [3H]-hypoxanthine supplemented media in triplicate at a concentration of 0.5 µCi [3H]-hypoxanthine per well in a total of 100 μL culture/well. Negative control growth wells containing 100 μM CQ were included on each plate in triplicate. Plates were incubated at 37°C in special gas for the remaining period of 72 h post-synchronization after which they were frozen at −80°C and harvested as described previously. The full method is graphically depicted in Fig. 3C.

### Data analysis

#### Hemozoin nucleation assay

Total hemozoin formed was calculated from heme quantification by spectroscopy as described above. Percent inhibition in drug treated samples was calculated as follows:


$$\%\;inhibition=1-\frac{nmols\;with\;drug}{avg\;nmols\;in\;no\;drug\;control}$$

Average percent inhibition values were plotted against drug concentrations in GraphPad Prism 10. IC_50_ values were calculated using the least-squares-regression model on the plotted data. 95% CI of each model and its IC_50_ values were also calculated using Prism. An extra-sum-of-squares *F*-test was performed to compare the statistical differences between IC_50_ values.

#### Hemozoin extension assay

Extension of the original 5 nmol β-hematin input into each reaction was calculated by first measuring the average amount of β-hematin recovered from the negative control samples. This value was subtracted from the positive control samples of β-hematin with heme to measure extension in the absence of drug inhibition. Percent inhibition in drug-treated samples was calculated as follows:


$$\%\;inhibition=1-\frac{\left(nmols\;with\;drug\right)-\left(nmols\;\beta \;hematin\;control\right)\;}{\left(nmols\;without\;drug\right)-\left(nmols\;\beta \;hematin\;control\right)}$$

IC_50_ values were calculated and statistically analyzed as described previously.

#### Drug inhibition assays

Percent inhibition was calculated from DPM measurements as follows:


$$\%\;inhibition=1-\frac{\left(DPM\;drug\;treated\;sample\right)-\left(avg\;DPM\;negative\;growth\;control\right)\;}{\left(avg\;DPM\;positive\;growth\;control\right)-\left(avg\;DPM\;negative\;growth\;control\right)}$$

Average percent inhibition values were plotted against drug concentrations for each stage. IC_50_ values were calculated and statistically analyzed as previously described.

#### IC_50_ determination

The least-squares regression model was used to plot a non-linear best-fit curve by minimizing the sum-of-squares of the vertical distances between each replicate data point and the curve up to 1,000 iterations. Assumptions include restriction of percent inhibition values between 0 and 100, and Gaussian distribution of the data.
